# The Role of Intermittent Hypoxia on the Proliferative Inhibition of Rat Cerebellar Astrocytes

**DOI:** 10.1371/journal.pone.0132263

**Published:** 2015-07-14

**Authors:** Sheng-Chun Chiu, Yu-Jou Lin, Sung-Ying Huang, Chih-Feng Lien, Shee-Ping Chen, Cheng-Yoong Pang, Jian-Hong Lin, Kun-Ta Yang

**Affiliations:** 1 Department of Research, Taichung Tzu Chi Hospital, Buddhist Tzu Chi Medical Foundation, Taichung, Taiwan; 2 Physiological and Anatomical Medicine, School of Medicine, Tzu Chi University, Hualien, Taiwan; 3 Department of Ophthalmology, Mackay Memorial Hospital, Hsinchu, Taiwan; 4 Institute of Medical Sciences, School of Medicine, Tzu Chi University, Hualien, Taiwan; 5 Tzu Chi Stem Cells Center, Hualien Tzu Chi Hospital, Buddhist Tzu Chi Medical Foundation, Hualien, Taiwan; 6 Department of Medical Research, Hualien Tzu Chi Hospital, Buddhist Tzu Chi Medical Foundation, Hualien, Taiwan; 7 PhD program in Pharmacology and Toxicology, Tzu Chi University, Hualien, Taiwan; 8 Department of Physiology, School of Medicine, Tzu Chi University, Hualien, Taiwan; University of Pecs Medical School, HUNGARY

## Abstract

Sleep apnea syndrome, characterized by intermittent hypoxia (IH), is linked with increased oxidative stress. This study investigates the mechanisms underlying IH and the effects of IH-induced oxidative stress on cerebellar astrocytes. Rat primary cerebellar astrocytes were kept in an incubator with an oscillating O_2_ concentration between 20% and 5% every 30 min for 1–4 days. Although the cell loss increased with the duration, the IH incubation didn’t induce apoptosis or necrosis, but rather a G0/G1 cell cycle arrest of cerebellar astrocytes was noted. ROS accumulation was associated with cell loss during IH. PARP activation, resulting in p21 activation and cyclin D1 degradation was associated with cell cycle G0/G1 arrest of IH-treated cerebellar astrocytes. Our results suggest that IH induces cell loss by enhancing oxidative stress, PARP activation and cell cycle G0/G1 arrest in rat primary cerebellar astrocytes.

## Introduction

Intermittent hypoxia (IH) is defined as repeated episodes of hypoxia interspersed with episodes of normoxia [1]. Although beneficial effects of IH pre-conditioning in subsequent lethal hypoxia in mice had been reported [2], the link between IH and several adverse events such as hypertension, developmental defects, neuropathological problems and sleep apnea syndrome have not been examined. Sleep apnea is a major public health problem because of its high prevalence and severe life-threatening consequences [3]. Obstructive sleep apnea (OSA), manifested as periodic decreases of arterial blood oxygen or intermittent hypoxia (IH), is the most prevalent type of sleep apnea. Patients with OSA have increased risk of cardiovascular diseases and neuro-cognitive deficits [4, 5]. Magnetic resonance imaging studies in OSA patients have revealed significant size-reductions in multiple sites of the brain, including the cortex, temporal lobe, anterior cingulated, hippocampus, and cerebellum [6].

Reoxygenation (therapy) of OSA increases the risk of oxidative stress and cell injury. Oxidative stress results primarily from excessive ROS, including superoxide (O_2_^−^‧), hydrogen peroxide (H_2_O_2_), and the hydroxyl radical (OH‧) [7]. Cells exposed to excessive oxidative stress are often subject to unfolded protein response, DNA damage and cell death. DNA damages usually results in, Poly (ADP-ribose) polymerase (PARP) activation, triggering the progression of the cell cycle to facilitate DNA repair [8, 9]. In case of severe DNA damage, the over-activation of PARP will lead to NAD^+^/ATP-depletion necrosis or AIF-mediated apoptosis [9, 10]. Increasing levels of ROS are also associated with the IH-induced CNS dysfunction.

Astrocytes are dynamic cells that maintain the homeostasis of CNS, and establish and maintain the CNS boundaries, including the blood-brain barrier (BBB) and the glial limitans, through interactions with endothelial and leptomeningeal cells, respectively [11]. Several reports have suggested that astrocytes promote remyelination and the formation of new synapses and neurons through the release of neurotrophic factors [12, 13]. Astrocytes (star-shaped cells) are involved in the physical structuring of the brain. They are the most abundant glial cells in the brain that are closely associated with neuronal synapses [14], and they regulate the transmission of electrical impulses within the brain. Glial cells are also involved in providing neurotrophic signals to neurons required for their survival, proliferation, and differentiation [15]. In addition, reciprocal interactions between glia and neurons are essential for many critical functions in brain health and disease. Glial cells play pivotal roles in neuronal development, activity, plasticity, and recovery from injury [16]. The idea that astrocytes have active roles in the modulation of neuronal activity and synaptic neurotransmission is now widely accepted [17].

This study evaluates the effects of IH-induced oxidative stress on rat cerebellar astrocytes cell loss, as well as the underlying pathways involved in these processes. We show ROS accumulation and PARP activation in IH-induced cell loss in rat cerebellar astrocytes. We further demonstrate PARP and p21 activation play roles in IH-induced cell cycle arrest and proliferation inhibition.

## Materials and Methods

### Chemicals and reagents

Basal modified Eagle’s medium, fetal calf serum, and gentamycin were purchased from Gibco (Carlsbad, CA). 2’,7’-dichlorodihydrofluorescein diacetate (DCFDA), DHE (Dihydroethidium) were purchased from Molecular Probes (Eugene, OR). The TUNEL kit was purchased from Roche Molecular Biochemicals (Mannhiem, Germany). All other chemicals were purchased from Sigma (Lt. Louis, MO).

### Primary cultures of rat cerebellar astrocytes

All procedures were performed in strict accordance with the recommendations in the Guide for the Care and Use of Laboratory Animals of the Tzu Chi University. The protocol was approved by the Institutional of Animal Care and Use Committee (IACUC) of the Tzu Chi University (Permit Number: 96062). All efforts were made to minimize animal suffering. In brief, astrocyte cultures were prepared from the cerebella of 7-day-old SD rats (of either sex), as described in our previous studies [18, 19]. The cerebellum was dissected and dissociated by mechanical chopping, and then trypsinized to obtain cell suspension. Cells were grown on 12 mm-diameter coverslips and maintained in 5% CO_2_-/95% humidified air at 37°C. The culture medium was basal modified Eagle’s medium (BMEM), supplemented with 10% fetal calf serum (FCS), and penicillin/streptomycin. Most of the cells remaining after 7 to 10 days of culturing of were astrocytes and were ready to be used for the following experiments.

### IH exposure

IH exposure was performed as described [20]. Cerebellar astrocytes were placed in Plexiglas box chambers (length 20 cm, width 20 cm, height 8 cm) and exposed to normoxia (RA; 20% O_2_, 5% CO_2_, and balance N_2_) or intermittent hypoxia (IH; 5% O_2_, 5% CO_2_, and balance N_2_ for 30 min alternating with 30-min to RA) using a timed solenoid valve controlled by DO-166MT-1SXS (Shelfscientific, USA) for 1–4 days. Oxygen levels in the chamber were continuously monitored using an oxygen detector.

### MTT assay

Cell viability after treatment with various conditions was evaluated using the MTT assay preformed in triplicate. Briefly, cells (2 x 10^5^/well) were incubated in a 3.5 cm petri dish containing 2 ml of serum-containing medium. Cells were allowed to adhere for 18–24 h and then were washed with phosphate-buffered saline (PBS). After treatment for the indicated condition, cells were washed with PBS, and culture medium containing 300 μg/ml MTT was added for 1 h at 37°C. After the MTT medium was removed, 2 ml of DMSO were added to each well. Absorbance at 570 nm was detected by a Multiskan EX ELISA Reader (Thermo Scientific, Rockford, IL). The absorbance for control group cells was considered to be 100%.

### Detection of cellular ROS

Intracellular levels of O_2_^–^• and H_2_O_2_ were detected respectively, using DHE or DCFDA. Both DHE and DCFDA are cell-permeable and become highly fluorescent on oxidation by O_2_^–^• or H_2_O_2_, respectively. Cells were loaded for 60 min at room temperature with 10 μM DHE or 5 μM DCFDA. The respective excitation/emission wavelengths for DHE and DCFDA were 488/610 nm and 488/540 nm. Signal increases are presented as the peak/basal fluorescence ratio (F/F_0_).

### Cell cycle analysis

The cell cycle was determined by flow cytometry using DNA staining dye to reveal the total amount of DNA. Cells were harvested with 0.25% trypsin/EDTA, then collected, washed with PBS, fixed with cold 70% ethanol for 1 h, and stained with a solution containing 20 μg/ml propidium iodide (PI), 0.2 mg/ml RNase A, and 0.1% Triton X-100 for 1 h in the dark. The cells were then analyzed using a FACScan flow cytometer (equipped with a 488-nm argon laser) to measure the DNA content. The data were obtained and analyzed with CellQuest 3.0.1 (Becton Dickinson, Franklin Lakes, NJ) and ModFitLT V2.0 software.

### Immunocytochemistry

Cells cultured on coverslips were treated with various conditions and fixed with cold 4% para-formaldehyde. The fixed cells were washed twice in PBS, and incubated in a cold permeabilization solution (0.15% Triton X-100) for 5 min. Cells were washed with PBS and incubated with 5% non-fat milk at room temperature for 10 min. First antibodies were incubated at 4°C overnight. The cells were washed with PBS three times and then incubated with FITC or TRITC-conjugated secondary antibody for 1 h at room temperature. The cells were then washed with PBS three times and counterstained with 300 nM Hoechst 33342 for 10 min. Images were obtained with a confocal microscope (TCS-SP, Leica).

### TUNEL assay

Cells were subjected to IH or RA for the indicated time and then examined for apoptosis using the TUNEL assay (*In Situ* Cell Death Detection Kit, Roche).

### Western blotting

Cells were lysed on ice with 200 μl of lysis buffer (50 mM Tris-HCl, pH 7.5, 0.5 M NaCl, 5 mM MgCl2, 0.5% Nonidet P-40, 1 mM phenylmethylsulfonyl fluoride, 1 μg/ml pepstatin, and 50 μg/ml leupeptin) and centrifuged at 10000 x g at 4°C for 10 min. The protein concentrations in the supernatants were quantified using a BSA Protein Assay Kit. Electrophoresis was performed on a NuPAGE Bis-Tris Electrophoresis System using 20 μg of reduced protein extract per lane. Resolved proteins were then transferred to PVDF membranes. Membranes were blocked with 5% non-fat milk for 1 h at room temperature and then probed with the appropriate dilution of primary antibodies at 4°C overnight: β-actin (chemicon). p21, cyclin D1 (cell signaling). After the PVDF membrane was washed three times with TBS/0.2% Tween 20 at room temperature, it was incubated with the appropriate secondary antibody (goat anti-mouse or anti-rabbit, 1:10000) and labeled with horseradish peroxidase for 1 h at room temperature. All proteins were detected using Western Lightning Chemiluminescence Reagent Plus (Amersham Biosciences, Arlington Heights, IL).

### Confocal microscopy

Cells were observed using a laser scanning confocal microscope (TCS-SP, Leica). Images were analyzed using the microscope’s bundled software.

### Statistics

The results of fluorescence measurements and cell proliferation experiments are expressed as the mean ± SEM. The t-test and one-way ANOVA with post-hoc test were performed to test differences between groups using SPSS 18.0 software (SPSS Taiwan Corp.). All tests were considered to be statistically significant when *p* < 0.05.

## Results

### Intermittent hypoxia (IH) accelerated the cell loss of rat cerebellar astrocytes in vitro

**Fig 1:**
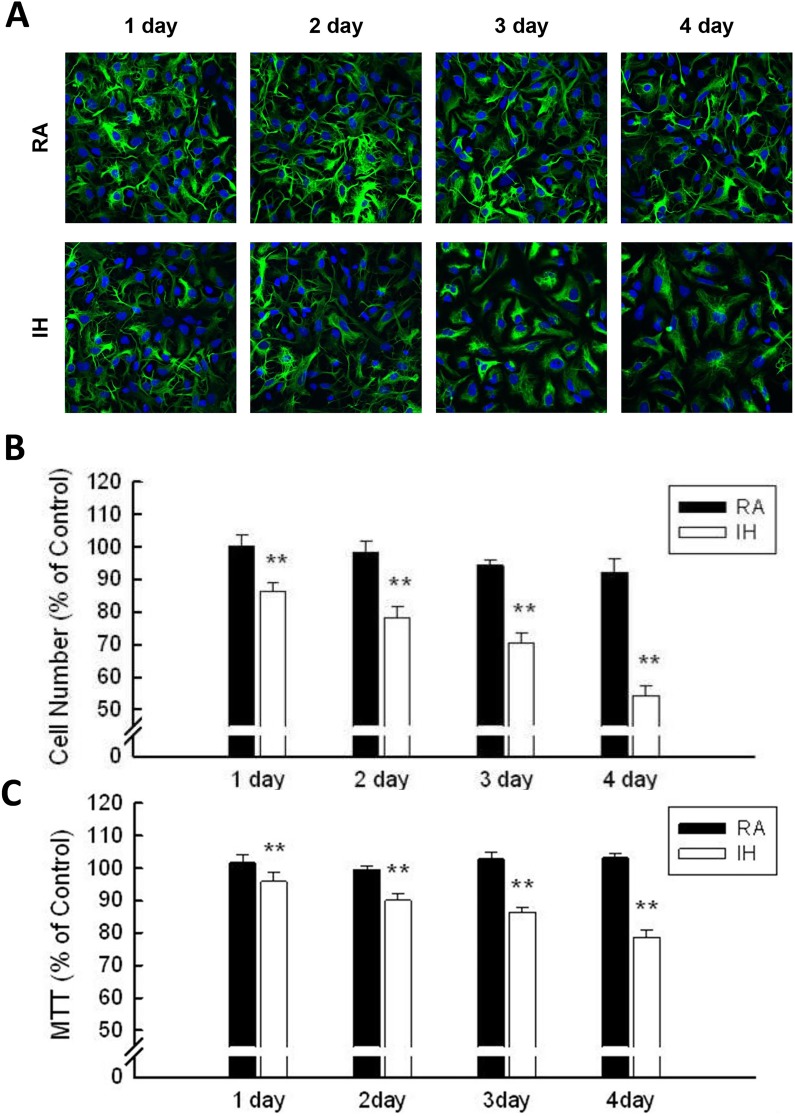
IH inhibits cell growth in rat cerebellar astrocytes. (A) Rat primary cerebellar astrocytes were incubated in an IH or RA chamber for 1~4 days followed by immuno-staining. Astrocytes were stained with anti-GFAP (green fluorescence), and nuclei were stained with Hoechst dye (blue fluorescence). (B) Double-stained cells were counted as astrocyte; the control group was set as 100%.(C) Astrocytes were incubated in an IH or RA chamber for 1~4 days and analyzed with MTT assay. Data are presented as means ± S.D. from three different experiments. **, *p* <0.01 versus control.

To elucidate the effects of intermittent hypoxia on rat cerebellar astrocytes, cells were cultured in an RA (normoxia) or IH (intermittent hypoxia) chamber for 1 to 4 days. After RA or IH incubation, cells were fixed and investigated by immuno-staining. Astrocytes were stained with anti-GFAP (Gilial fibrillay acidic protein, green fluorescence), and nuclei were stained with Hoechst dye (blue fluorescence) (Fig 1A). The cell number of astrocytes cultured in 5% CO_2_ on day 0 was set at as 100%. RA1~RA4 respectively represent the cell counts of astrocytes in normoxia from days 1 to 4. IH1~IH4 respectively represent the cell counts of astrocytes in IH from days 1 to 4. The cell numbers of RA1~RA4 were 100.38 ± 3.40%、98.19 ± 3.74%、94.36 ± 1.72%、92.29 ± 3.96%, respectively, while those of IH1~IH4 were 86.21 ± 2.69%、78.27 ± 3.28%、70.42 ± 3.26%、54.28 ± 3.19%, respectively (Fig 1B). Similar results were obtained by MTT assay (Fig 1C; RA1~RA4: 101.67 ± 2.38%, 99.5 ± 1.06%, 102.86 ± 2.14% and 103.27 ± 1.42%, respectively. IH1~IH4: 95.91 ± 2.84%, 90.09 ± 2.01%, 86.35 ± 1.36% and 78.73 ± 2.07%, respectively.) Taken together, these data suggest that IH incubation caused cell loss in rat primary cerebellar astrocytes.

### Cell loss due to IH was not related to apoptosis or necrosis in rat cerebellar astrocytes

**Fig 2:**
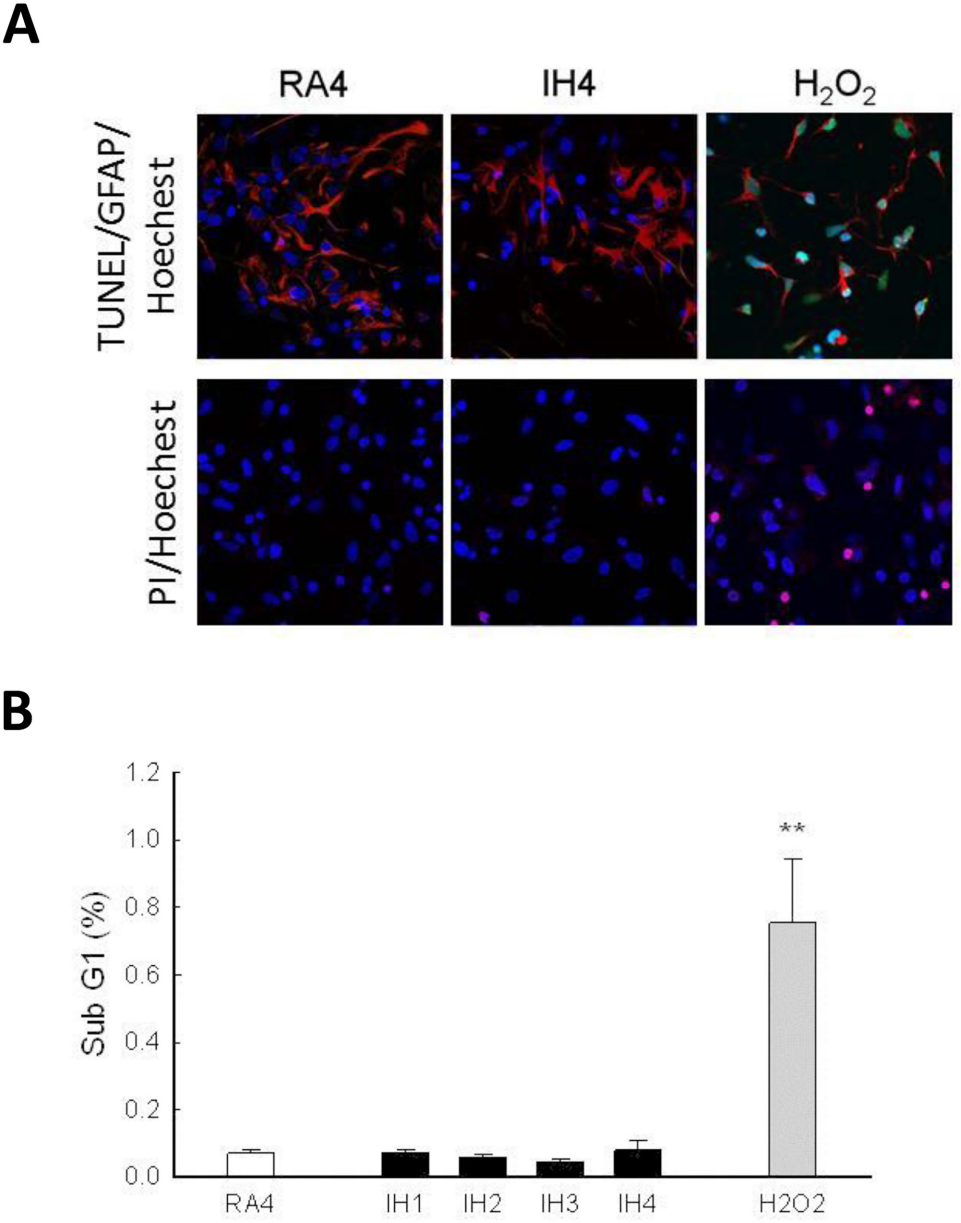
Cell loss due to IH was not related to apoptosis or necrosis. (A) Astrocytes were incubated in IH, RA or 100 μM H_2_O_2_ for 4 days and then subjected to TUNEL assay (upper panel) or PI staining (lower panel). Cell nuclei were stained with Hoechst dye (blue fluorescence). (B) Sub-G1 portion of the RA4, IH1~IH4 and H_2_O_2_ groups were analyzed by flow cytometer. Data are presented as means ± S.D. from three different experiments. **, *p* <0.01 versus vehicle.

To clarify the roles of apoptosis and necrosis in IH-induced cell loss, astrocytes were incubated in RA or IH chambers for 4 days and analyzed using the TUNEL assay and PI immuno-staining (Fig 2A). Astrocytes treated with 100 μM H_2_O_2_ were used as the positive control. There were no TUNEL-positive (green fluorescence) cells in the RA4 or IH4 groups as compared to the H_2_O_2_ group (Fig 2A, upper panel). There were also no PI-positive (red fluorescence) cells in RA4 or IH4 group as compared to the H_2_O_2_ group (Fig 2A, lower panel). In addition, when the sub-G1 cells of various treatments were analyzed using flow cytometry, no significant increase of the sub-G1 cell population was found in the RA4 or IH4-treated groups (Fig 2B). These results suggest that IH incubation-induced cell loss didn’t correlate with apoptosis or necrosis induction in rat cerebellar astrocytes.

### IH induced G0/G1 phase arrest in rat cerebellar astrocytes

**Fig 3:**
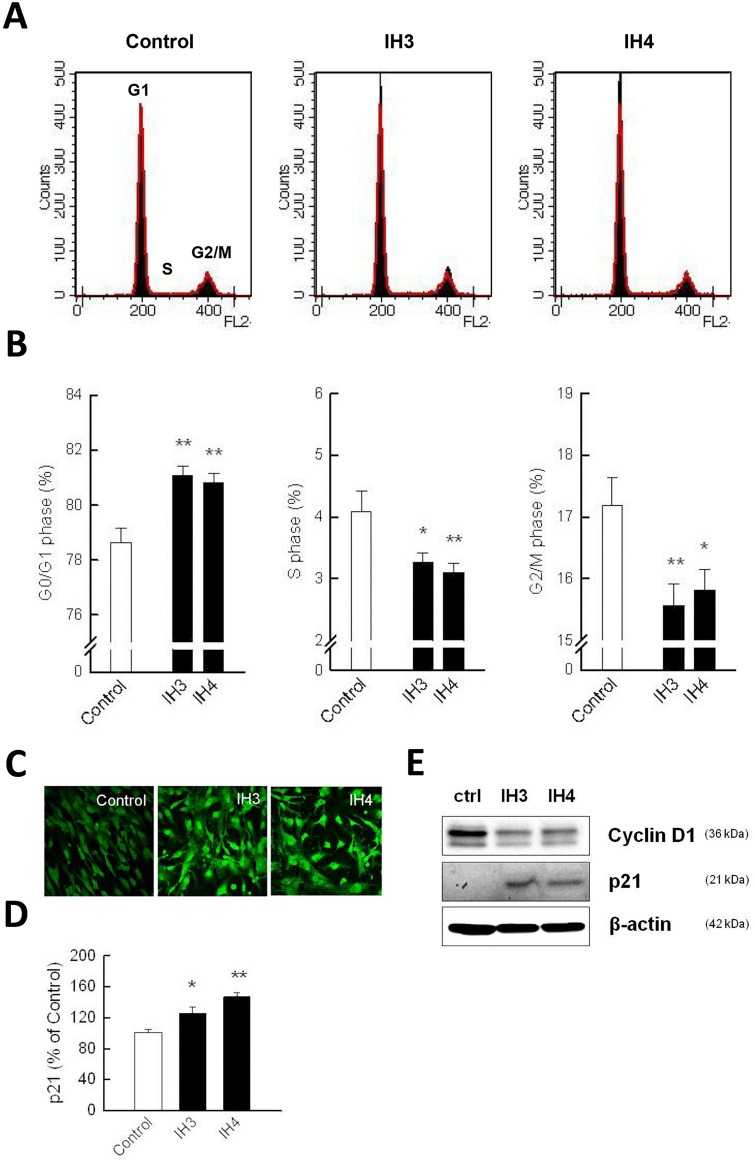
IH-induced cell loss was associated with cell cycle G0/G1 arrest. (A) The cell cycle profiles of the control, IH3 and IH4 groups were analyzed by flow cytometry. (B) IH-induced G0/G1 arrest significantly increased at IH3 (81.09 ± 0.33%) and IH4 (80.82 ± 0.35%) as compared to the control (78.64 ± 0.54%). (C) Immuno-staining for p21 in the control, IH3 and IH4 groups. (D) Quantitative assessment of immuno-staining data from Fig 3C. (E) Western blot analysis of cyclin D1 and p21 was performed in the control, IH3 and IH4 groups. β-actin was used as an internal control. Data are presented as means ± S.D. from three different experiments. *, *p* <0.05; **, *p* <0.01 versus vehicle.

The effect of IH on cell cycle progression was also examined: Flow cytometric analysis showed that IH resulted in the accumulation of cells in G0/G1 phase arrest (Fig 3A). Treatment of cells with IH for 3 and 4 days increased the percentage of cells in the G0/G1 phase to 81.09 ± 0.33% and 80.82 ± 0.35%, respectively, as compared to the control group (78.64 ± 0.54%, Fig 3B). To further examine the underlying mechanism of the G0/G1 arrest caused by IH, the expression of the cell cycle regulatory protein, p21, was examined. Immuno-staining showed that the fluorescence intensity of p21 was increased higher in the IH groups (both IH3 & IH4) than in the RA control (Fig 3C). The relative intensities of the fluorescence were IH3 (125.40 ± 8.01%) and IH4 (146.73 ± 5.68%), as compared to the RA control astrocytes (Fig 3D). The upregulation of p21 by IH in astrocytes was further validated by western blotting. We also found that the upregulation of p21 concurred with the inhibition of cyclin D expression (Fig 3E). These results suggest that IH induced cell cycle G0/G1 arrest and might be associated with the activation of p21 in rat cerebellar astrocytes.

### IH induced ROS accumulation in rat cerebellar astrocytes *in vitro*

**Fig 4:**
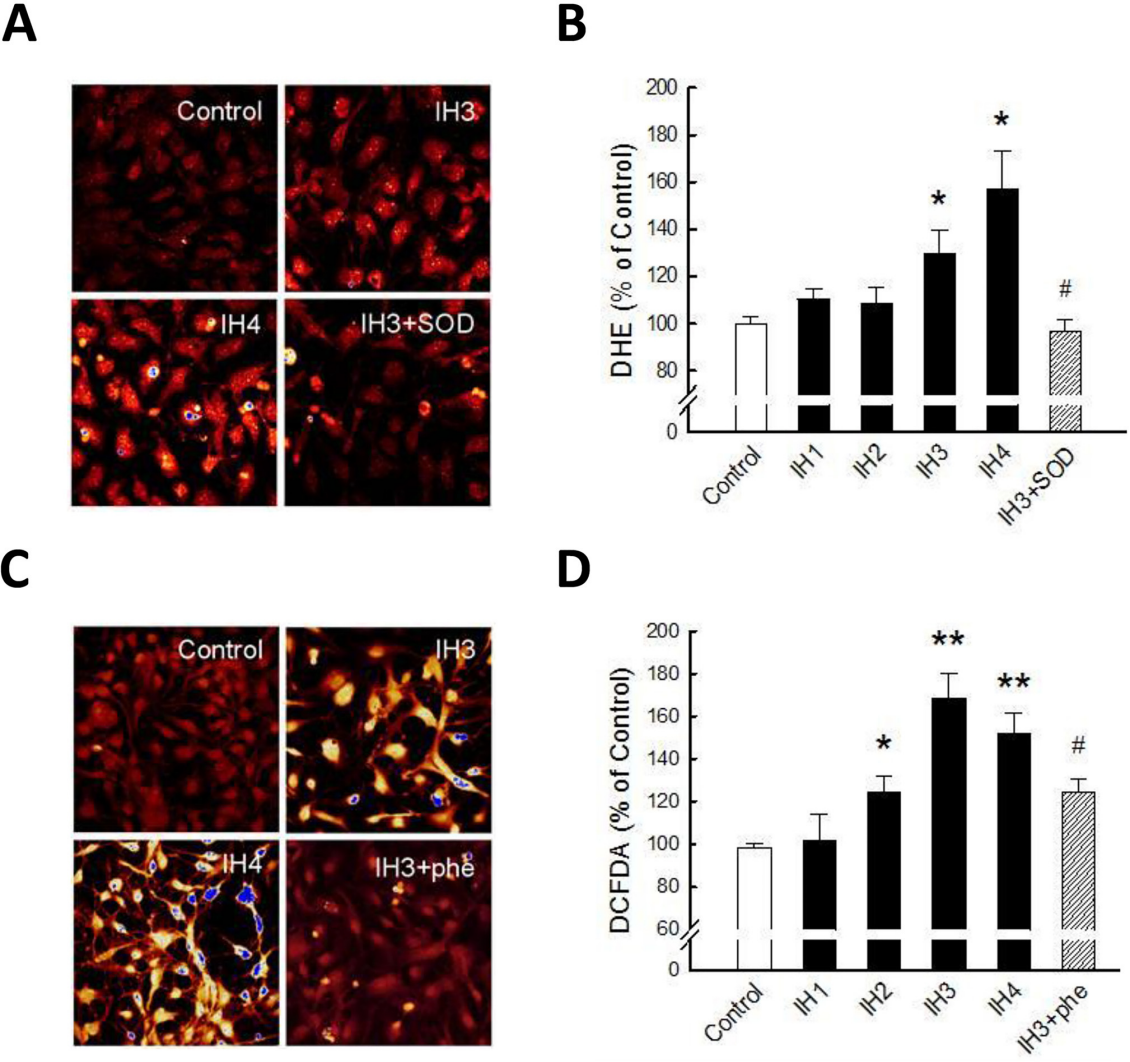
IH induced ROS accumulation in rat cerebellar astrocytes. (A) DHE staining was performed for detection of O_2_^−‧^ in astrocytes incubated in the control, IH3, IH3+SOD and IH4 groups. (B) Quantitative assessment of immuno-staining data from Fig 4A. (C) DCFDA staining was performed for the detection of OH^‧^ astrocytes incubated in the control, IH3, IH4, H_2_O_2_, IH3+phe and IH4+phe groups. (D) Quantitative assessment of immunostaining data from Fig 4C. Data are presented as means ± S.D. from three different experiments. *, *p* <0.05, **, *p* <0.01 versus control; ^##^ *p* <0.01 versus IH groups.

Previous studies have suggested that IH may increase ROS in experimental animals and in cell cultures. To further investigate the role of ROS in IH-induced astrocytic cell loss, we first examined the O_2_^–^• level after IH incubation. Astrocytes were incubated in IH condition for number of days indicated in Fig 4A and stained with DHE to detect intracellular O_2_^–^• (Fig 4A). Co-treatment of the astrocytes with 5U/ml superoxide dismutase (SOD) enhanced the O_2_^–^•to H_2_O_2_, thus reducing the IH-induced fluorescence intensity (IH3+SOD group in Fig 4A lower panel). The respective average fluorescence intensities of DHE staining in IH1 to IH4 groups were 110.4 ± 4.23%, 108.83 ± 6.75%, 129.42 ± 10.21%, and 157.22 ± 16.07%. The fluorescence intensity significantly decreased to 97 ± 4.48% after SOD co-treatment (Fig 4B). OH• level were also examined by DCFDA staining. The fluorescence intensities of the IH3 and IH4 groups were higher than in the control group (Fig 4C). Co-treatment with 100 nM 1,10-Phenanthroline (Phe) decreased the ROS generation in IH3 group (Fig 4C lower panel). The fluorescence intensities increased to 168.44 ± 11.82% and 151.69 ± 9.59% (IH3 and IH4, respectively) and significantly decreased to 124.33 ± 5.99% after Phe co-treatment (Fig 4D). These data suggest that IH induced ROS (O_2_^−^• and- OH•) accumulation in rat cerebellar astrocytes *in vitro*.

### IH induced PARP activation in rat cerebellar astrocytes *in vitro*

**Fig 5:**
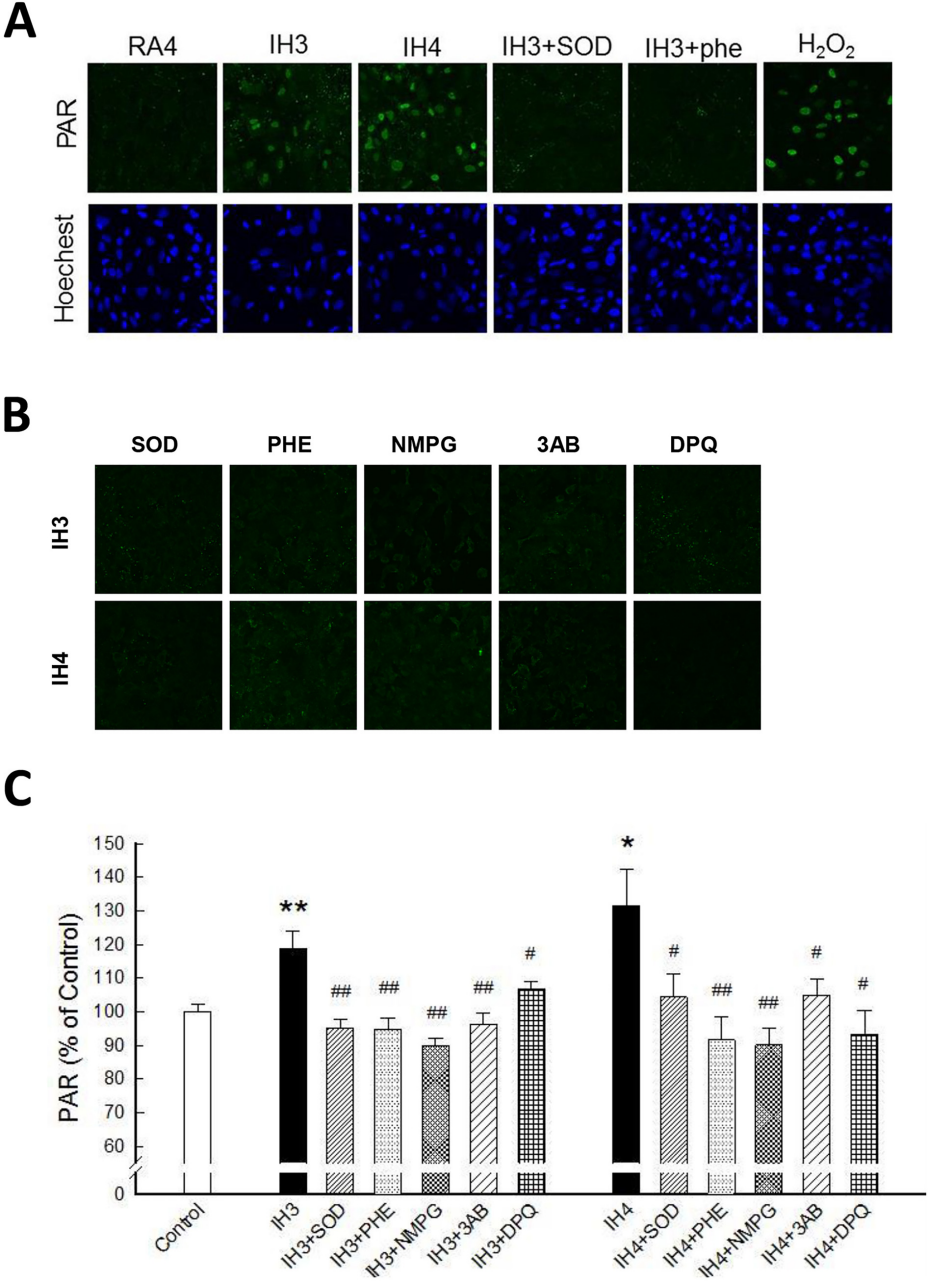
IH induced ROS-mediated PAR expression in rat cerebellar astrocytes. (A) Immuno-staining for PAR expression in RA3, IH3, IH4, IH3+SOD, IH3+phe and H_2_O_2_ groups. (B) Immuno-staining for PAR expression in IH3 and IH4 groups treated with ROS or PARP inhibitors. (C) Quantitative assessment of immuno-staining data from Fig 5A and B. *, *p* <0.05, **, *p* <0.01 versus control; ^#^, *p* <0.05, ^##^ *p* <0.01 versus IH groups.

Excessive accumulation of ROS induces oxidative stress and leads to DNA damage. Poly (ADP-ribose) polymerase (PARP) is a family of proteins activated by DNA damage and apoptosis. PARP usually attaches to regions of damaged DNA and catalyzes the synthesis of poly (ADP-ribose) (PAR) chains to itself and adjacent nuclear proteins. PAR thus serves as a signal for other DNA repair enzymes. To elucidate the roles of ROS and DNA damage in the IH-induced cell loss of rat cerebellar astrocytes, we investigated the expression PAR in the nuclei of IH-treated astrocytes. The fluorescence intensity of the PAR chains was dramatically increased in the IH group as compared to the control group (Fig 5A). Astrocytes treated with 100 μM H_2_O_2_ served as the positive control. Co-treatment with the PARP inhibitor 3-Aminobenzamide (3-AB, 1 mM) and DPQ (1 mM) diminished the IH-induced PAR expression (Fig 5B). The fluorescence intensity was quantified and compared to the control (Fig 5C): IH3+3-AB (96.31 ± 3.12%), IH3+DPQ (106.78 ± 2.06%), IH4+3AB (105 ± 4.86%), and IH4+DPQ (93.29 ± 7.15%).

To further verify that the IH-induced ROS accumulation activated the PAR expression, astrocytes were incubated in a medium containing-SOD (5U/ml), PHE (1 mM) or N-2-Mercaptopropionyl glycine (NMPG, 200 μM), respectively, during IH. The immuno-staining of PAR indicated that co-treatment with ROS inhibitor decreased the PAR expression as compared to IH group (Fig 5A and 5B). The fluorescence intensity was quantified and compared to the control (Fig 5C): IH3+ SOD (95.10 ± 2.78%), IH3+ PHE (94.77 ± 3.31%), IH3+ NMPG (89.79 ± 2.44%), IH4+ SOD (104.29 ± 6.81%), IH4+ PHE (91.57 ± 6.96%), and IH4+ NMPG (90.14 ± 4.84%). These data suggest that IH induced PARP activation and caused PAR polymerization in rat cerebellar astrocytes.

### IH-induced cell loss can be inhibited by anti-oxidants or PARP-inhibitors

**Fig 6:**
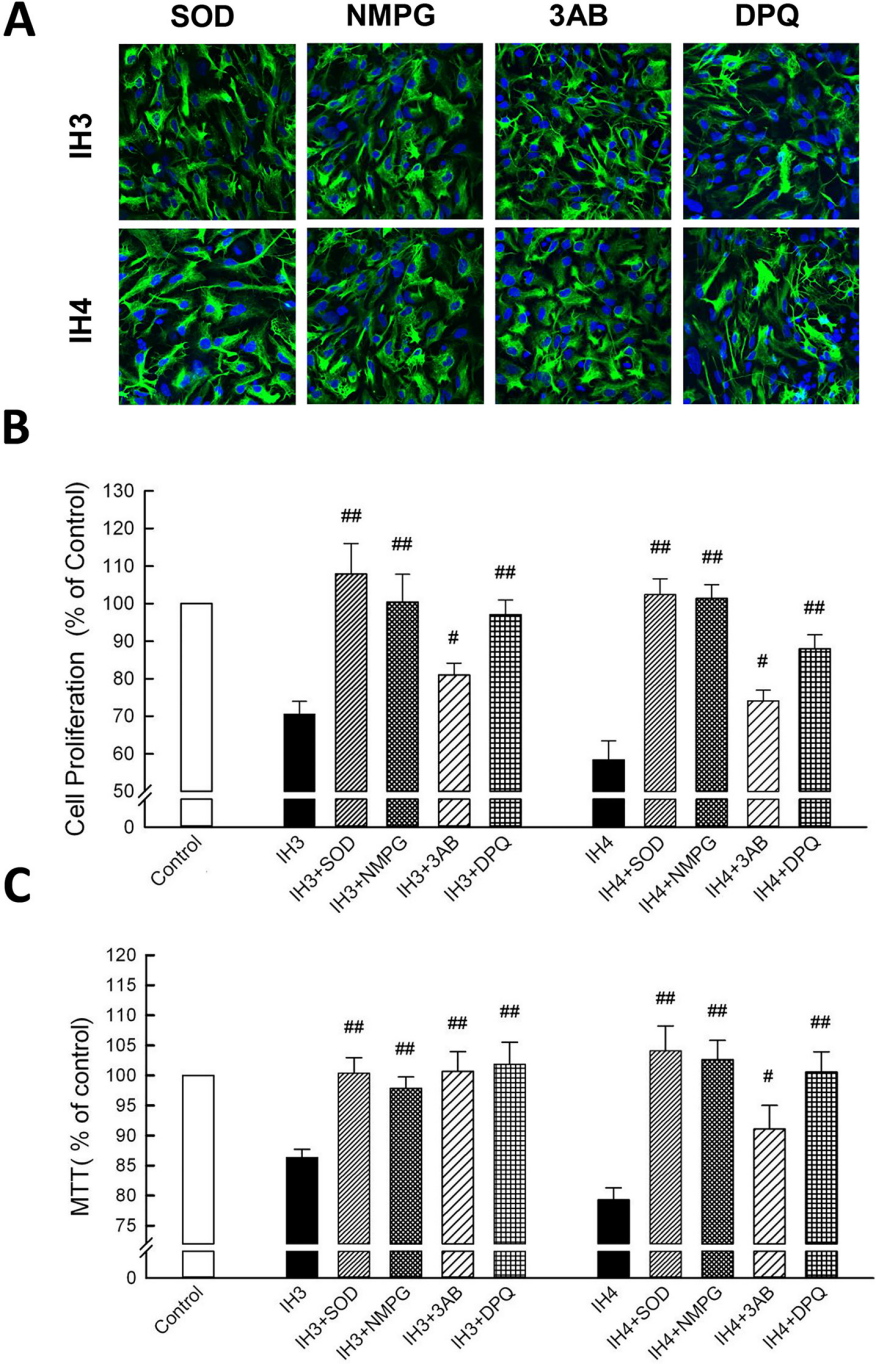
IH-induced cell loss can be inhibited by ROS- or PARP-inhibitors treatment. (A) Astrocytes cultured in IH3 and IH4 and treated with ROS or PARP inhibitors groups were stained with anti-GFAP (green fluorescence), and the nuclei were stained with Hoechst dye (blue fluorescence). (B) Double-stained cells were counted as astrocyte; the control group was set as 100%. (C) Astrocytes were incubated in an IH chamber treated with ROS or PARP inhibitors then subjected to MTT assay. Data are presented as means ± S.D. from three different experiments. ^#^, *p* <0.05, ^##^, *p* <0.01 versus control.

To clarify the roles of ROS and PARP activation in IH-induced astrocytic cell loss, cells were cultured at IH with ROS or PARP inhibitors. Astrocytes were stained with anti-GFAP (green fluorescence), and nuclei were stained with Hoechst dye (blue fluorescence) (Fig 6A). Cells cultured in 5% CO_2_ on day 0 were set at as 100%. The double-stained cells were recognized as astrocytes and cell counting showed IH3 (70.54 ± 3.37%), IH3+ SOD (100.38 ± 2.58%), IH3+ NMPG (97.86 ± 1.9%), IH3+3AB (100.69 ± 3.27%), IH3+DPQ (101.85 ± 3.68%), IH4 (79.31 ± 1.98%), IH4+SOD (104.1 ± 4.14%), IH4+NMPG (102.64 ± 3.19%), IH4+3AB (91.1 ± 3.92%), and IH4+DPQ (100.57 ± 3.37%) (Fig 6B). Similar results were obtained by MTT assay analysis. Astrocytes were rescued by treatment with ROS or PARP inhibitor in IH incubation and compared to the control group: IH3 (86.35 ± 1.36%), IH3+SOD (100.38 ± 2.58%), IH3+NMPG (97.86 ± 1.9%), IH3+3AB (100.69 ± 3.27%), IH3+DPQ (101.85 ± 3.68%), IH4 (79.31 ± 1.98%), IH4+SOD (104.1 ± 4.14%), IH4+NMPG (102.64 ± 3.19%), IH4+3AB (91.10 ± 3.92%), and IH4+DPQ (100.57 ± 3.37%) (Fig 6C). Taken together, IH-induced astrocytic cell loss was correlated with ROS accumulation and PARP activation. In addition, treatment with ROS or PARP inhibitors restored the IH-induced cell loss in rat cerebellar astrocytes.

### IH-induced cell cycle arrest was inhibited by anti-oxidants or PARP-inhibitors

**Fig 7:**
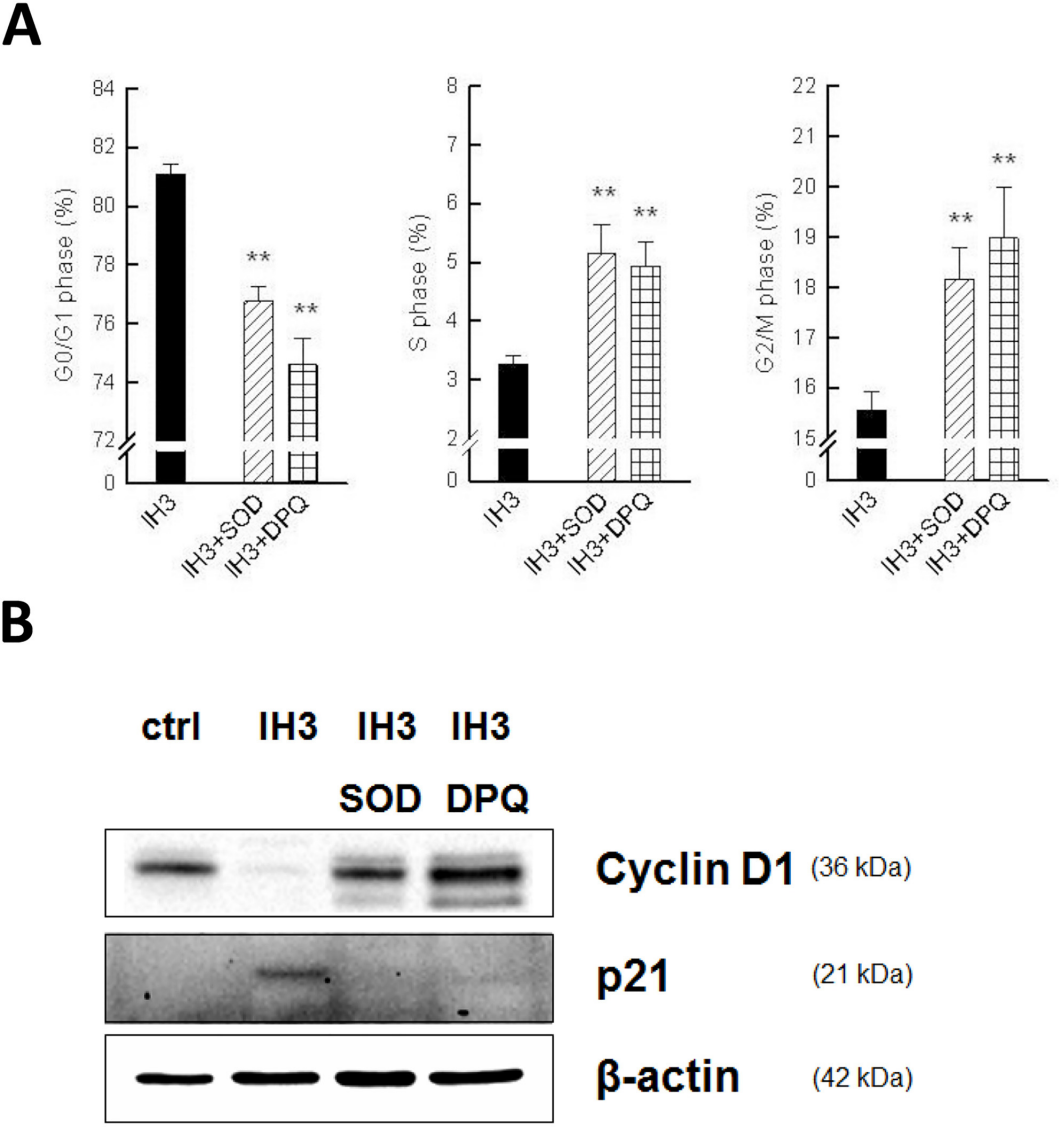
IH-induced cell cycle arrest can be inhibited by ROS- or PARP-inhibitor treatment. (A) Treatment with SOD or DPQ significantly decreased IH-induced G0/G1 arrest from 81.09 ± 0.33% to 76.57 ± 0.75% and 73.93 ± 1.17% (IH3+SOD and IH3+DPQ, respectively). (B) Western blot analysis of cyclin D1 and p21 was performed in the indicated groups. β-actin was used as an internal control. Data are presented as means ± S.D. from three different experiments. *, *p* <0.05; **, *p* <0.01 versus IH3.

Since IH-induced astrocytic cell loss was rescued by ROS or PARP inhibitors, we further examined their cell cycle profiles after SOD or DPQ treatment. Flow cytometric analysis showed that treatment with SOD or DPQ significantly inhibited IH-induced G0/G1 arrest in astrocytes: IH3 (81.09 ± 0.33%), IH3+SOD (76.75 ± 0.52%), and IH3+DPQ (74.59 ± 0.9%) (Fig 7A). The G0/G1 regulatory proteins were also examined by western blot analysis. ROS or PARP inhibitors increased the expression level of cyclin D1 and decreased the expression level of p21 (Fig 7B). These results suggest that IH induced cell cycle G0/G1 arrest in rat cerebellar astrocytes through ROS accumulation and PARP activation, and can be partially rescued by ROS or PARP inhibitors.

## Discussion

Our results demonstrate that IH induced oxidative stress in rat cerebellar astrocytes and led to cell loss *in vitro*. Several previous reports had indicated that chronic IH could elevate oxidative stress and increase apoptosis in mice cortical neurons [21, 22]. We previously reported that IH-induced oxidative stress and cell death in rat cerebellar granule cells [20]. However, in rat cerebellar astrocytes, we demonstrated that IH induced G0/G1 cell cycle arrest but not apoptosis or necrosis.

We also clarified that cyclin D1 is down-regulated and p21 is up-regulated after IH treatment. Recent studies have revealed that oxidative stress can cause cyclin D1 depletion [7] and cell cycle arrest can serve as a protective mechanism to reduce genotoxic damages from oxidative stress. The expression of cyclin D1 represents an important marker for assessing the integration of proliferative and growth inhibitory effects of oxidants on the redox-dependent signaling events that control cell cycle progression [23]. Reduction in the intracellular levels of ROS by ROS inhibitors can lead to increased expression of cyclin D1, entry in to the G1 phase and progression into the S phase. The p21 protein binds to and inhibits cyclin E/A-CDK2 and cyclin D-CDK4 complexes. The increase in p21 following IH treatment indicated that p21 might play an important role in inhibiting cell cycle progression. Both PARP and ROS inhibitors reduced the expression of p21 and restored the cell cycle progression.

IH-induced oxidative stress can lead to DNA damage, PARP activation, and PAR polymerization. Inhibition of oxidative damage by ROS inhibitors reduced PAR polymerization and restored the cell loss induced by IH. Treatment with a PARP inhibitor partially rescued cell loss after IH, and restored the expression of cyclin D1, as well as the progression of the cell cycle. These data suggested that PARP activation was involved in IH-induce astrocytic cell cycle arrest.

The cellular oxidation and reduction environment is influenced by the production and removal of ROS [7]. Thus, cellular ROS level could regulate cellular processes including cell proliferation and differentiation. The roles of astrocytes during ischemic injury has recently attracted considerable attention [24]. Astrocytes promote neuronal survival during ischemia by limiting neuronal damage and cell death caused by ROS [25, 26], excitotoxins [27] and other stressors. The glutathione system is responsible for the rapid clearance of organic hydroperoxides by astrocytes, along with the defense of the neurons against ROS [28, 29].

According to the astrocyte-neuron lactate shuttle hypothesis, astrocytes play a major role in supplying neurons with energy in the form of lactate [30, 31]. Monocarboxylate transporter 4 (MCT4) is expressed specifically in astrocytes and is involved in this process. Recent studies have suggested that IH preconditioning can provide protection to neurons against epilepsy through the upregulation of MCT4 expression in astrocytes *in vitro* and *in vivo* [32]. The expression level of MCT4 by astrocytes is controlled by oxygen tension via a HIF-1α-dependent mechanism [33]. It has been reported that PARP-1 inhibition reduced the transcriptional activity of HIF-1α [34]. Our results showed that IH-induced astrocyte cell loss can be rescued by PARP inhibition, however, the MCT4-mediated neuro-protection effect might be diminished by PARP inhibition, and this should be examined in future research.

## Conclusion

**Fig 8:**
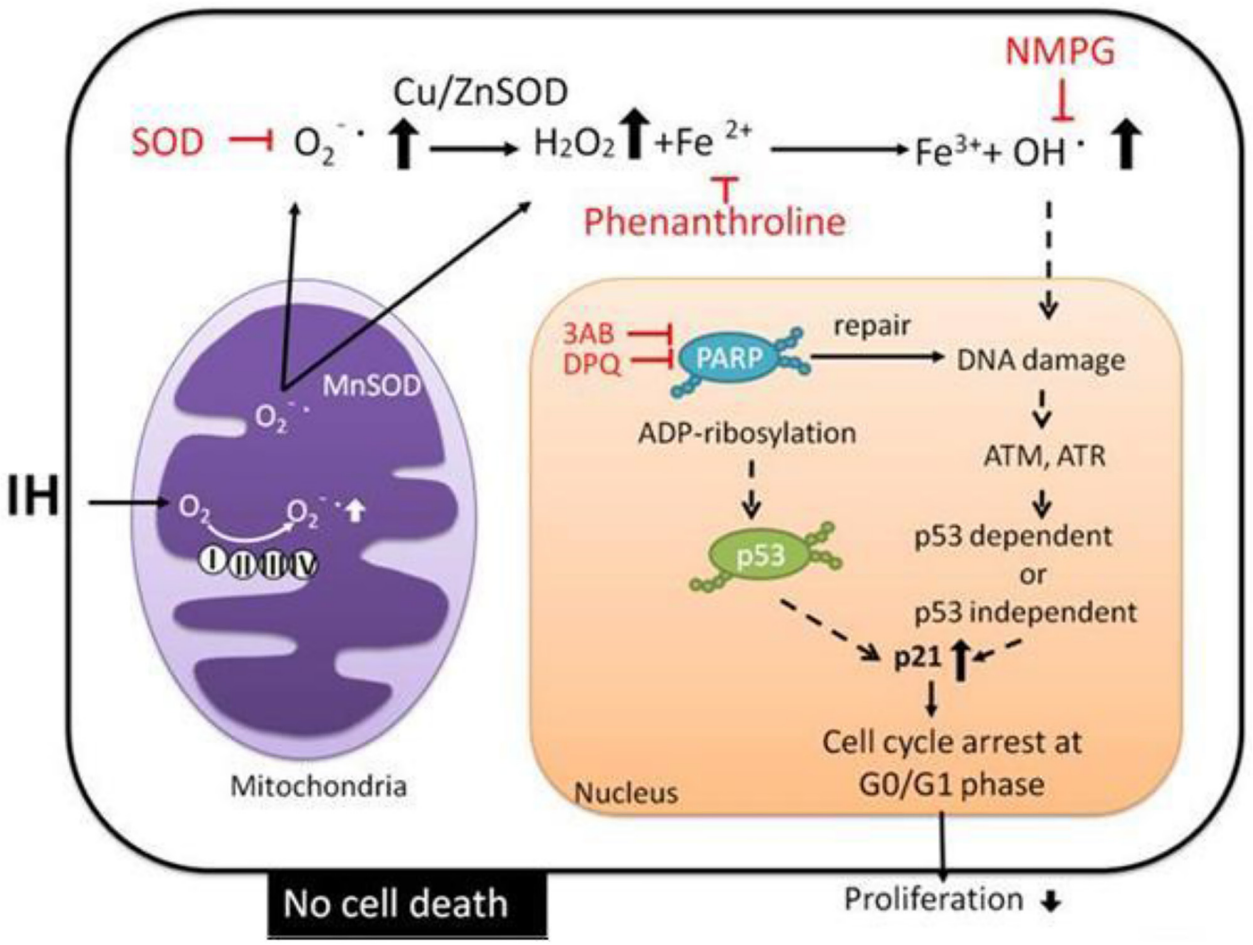
Possible molecular mechanisms for IH-induced cell loss in rat cerebellar astrocytes. IH incubation caused the accumulation of ROS (O_2_^−‧^and OH^‧^). Increasing oxidative stress induces DNA damage, leading to PARP activation and p21 activation, which induce cell cycle arrest in the G0/G1 phase.

Our study demonstrated the roles of ROS accumulation and PARP activation in IH-induced cell loss in rat cerebellar astrocytes. IH incubation causes ROS accumulation, PARP and p21 activation, and can subsequently lead to cell cycle G0/G1 arrest and proliferation inhibition (Fig 8).
